# The mitochondrial genome of *Homoneura picta* (Diptera: Lauxaniidae) and its phylogenetic analysis

**DOI:** 10.1080/23802359.2024.2333560

**Published:** 2024-06-24

**Authors:** Yao Yao, Yedi Shi, Keli Feng, Jiaoyi Du, Yiming Chang, Yaoyao Xue, Wenliang Li

**Affiliations:** College of Horticulture and Plant Protection, Henan University of Science and Technology, Luoyang, China

**Keywords:** Mitochondrial genome, Lauxaniidae, Homoneurinae, *Homoneura picta*

## Abstract

*Homoneura picta* belongs to the Homoneurinae subfamily of Lauxaniidae, and it is widely distributed and common in China. This study reports the newly sequenced mitochondrial genome of *H. picta*. The sequence is 15,469 bp long and contains 37 genes (13 protein-coding, 22 tRNA, and 2 rRNA genes) and a control region. The overall base composition is 38.4% for A, 37.7% for T, 14.1% for C, and 9.8% for G, with a bias toward A + T (76.1%). Phylogenetic analysis show that *Homoneura* is a sister genus of *Cestrotus*. We have successfully sequenced the mitochondrial genome of *H. picta*, which can be useful in investigating the phylogenetic status of Homoneurinae. Our results provide data for further studies of phylogeny in Diptera.

## Introduction

*Homoneura* Wulp (Diptera, Lauxaniidae) is one of the most species-rich genera of the subfamily Homoneurinae and the family Lauxaniidae, and consists of over 700 species globally. It is mainly distributed in the Palearctic, Neoearctic, Oriental, Australian, and African tropical zones, but is absent in neotropical zones. In China, 224 *Homoneura* Wulp species have been identified (You et al. 2023). Currently, relevant analysis has not been conducted for *Homoneura* because of the deficiency of mitochondrial genome data, only eight mitochondrial genomes from Lauxaniidae and two mitochondrial genomes from *Homoneura*, are known. *Homoneura picta* (de Meijere 1893) is widely distributed and commonplace in China. The spots on its wings are complex and varied. This study is the first to sequence the mitochondrial genome of *H. picta* and provide an essential DNA molecular resource for further investigations, which can be useful in exploring the phylogeny of Homoneurinae.

## Materials and methods

The *H. picta* (de Meijere 1893) specimens were collected on 6 October 2021 from Jingning in Lishui of Zhengjiang Province (27°43'N, 119°36'E), China. Ethical approval or permissions for sample collection were not required because the sample was an insect. The specimen was observed with a Motic SMZ-168 anatomic mirror. Genitalia preparation was made by removing and macerating the apical portion of the abdomen in cold saturated NaOH for 6 h, then rinsing and neutralizing it for dissection and study. After examination in glycerin, it was transferred to fresh glycerin and stored in a microvial pinned below the specimen. The specimen was identified by Xulong Chen. *H. picta* was distinguished from other species based on the following characteristics: arista was short pinnate, palpus brown, scutal black brown; a grey powder spot in the center of the base half; a yellow spot on both sides of the end half; legs yellow, all femurs end 1/4 yellow (Sasakawa 1992). The specimens were preserved in 100% absolute ethanol and deposited at the Henan University of Science and Technology, Henan, China (www.haust.edu.cn, contact person: Yao Yao; e-mail: 210321261426@stu.haust.edu.cn) under the voucher number Lau202000002.

**Figure 1.**
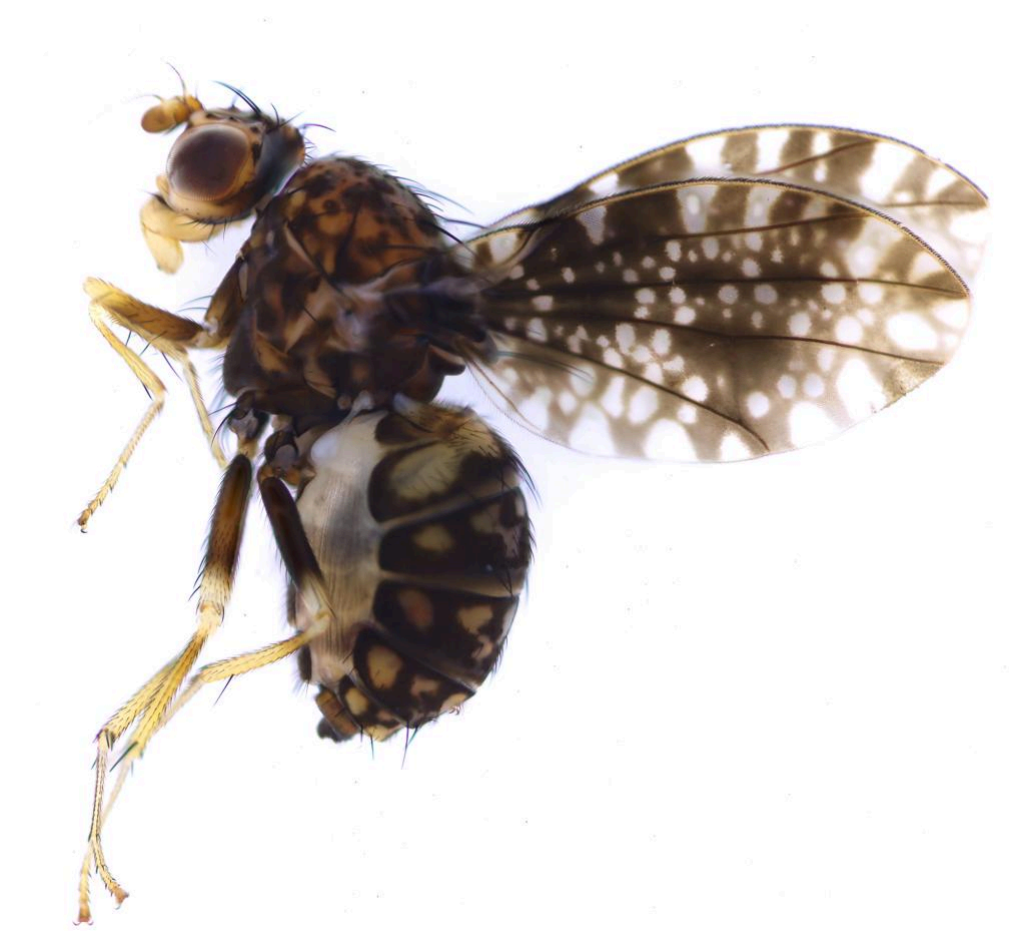
The specimen map of *H. picta*. This species reference image was taken by Yao.

The photograph of *H. picta* habitus was taken using a Canon EOS6D microscope (Canon, Tokyo, Japan) and stacked using HELICO FOCUS v7.0.2.0(Helicon Soft, Kharkiv, Ukraine) (Figure 1). Total genomic DNA was extracted from the whole body (except the head and wings) using the TIANamp Micro DNA Kit (Tiangen, Beijing, China) and stored at −20 °C. The mitochondrial genome was sequenced by Sang on Biotech Co., Ltd. (Shanghai, China). The purified DNA was fragmented and used to construct short-insert libraries (insert size 430 bp) using Illumina TruSeq Nano DNA Library Preparation Kit and then sequenced on the Illumina NovaSeq 6000 platform. Approximately 4 GB of high-quality data was used to assemble the mitochondrial genome using the de novo assembler IDBA-UD (Peng et al. 2012). The bait sequence (*COI* gene) was amplified *via* standard PCR. Gene annotation was performed using the MITOS2 webserver (Donath et al. 2019). The protein-coding genes (PCGs) were identified manually, using Geneious 9.0.2, by aligning them with other species from the closely related Lauxaniidae family. A circular map was created using CGView (Grant and Stothard 2008).

Phylogenetic analysis was conducted using IQ-tree, based on 13 PCGs and 2 ribosomal genes from *H. picta* and 15 other sequences downloaded from GenBank, including seven Lauxanioidea species, one Tephritoidea specie, one Sciomyzoidea specie, one Ephydroideea specie, one Muscoidea specie, one Oestroidea specie, one Phoroidea specie, one Opomyzoidea specie, one Syrphoidea specie. Sequence alignment and alignment trimming were performed using MEGA11, while the maximum-likelihood method was used to rebuild the phylogenetic tree using IQ-tree.

## Results

**Figure 2.**
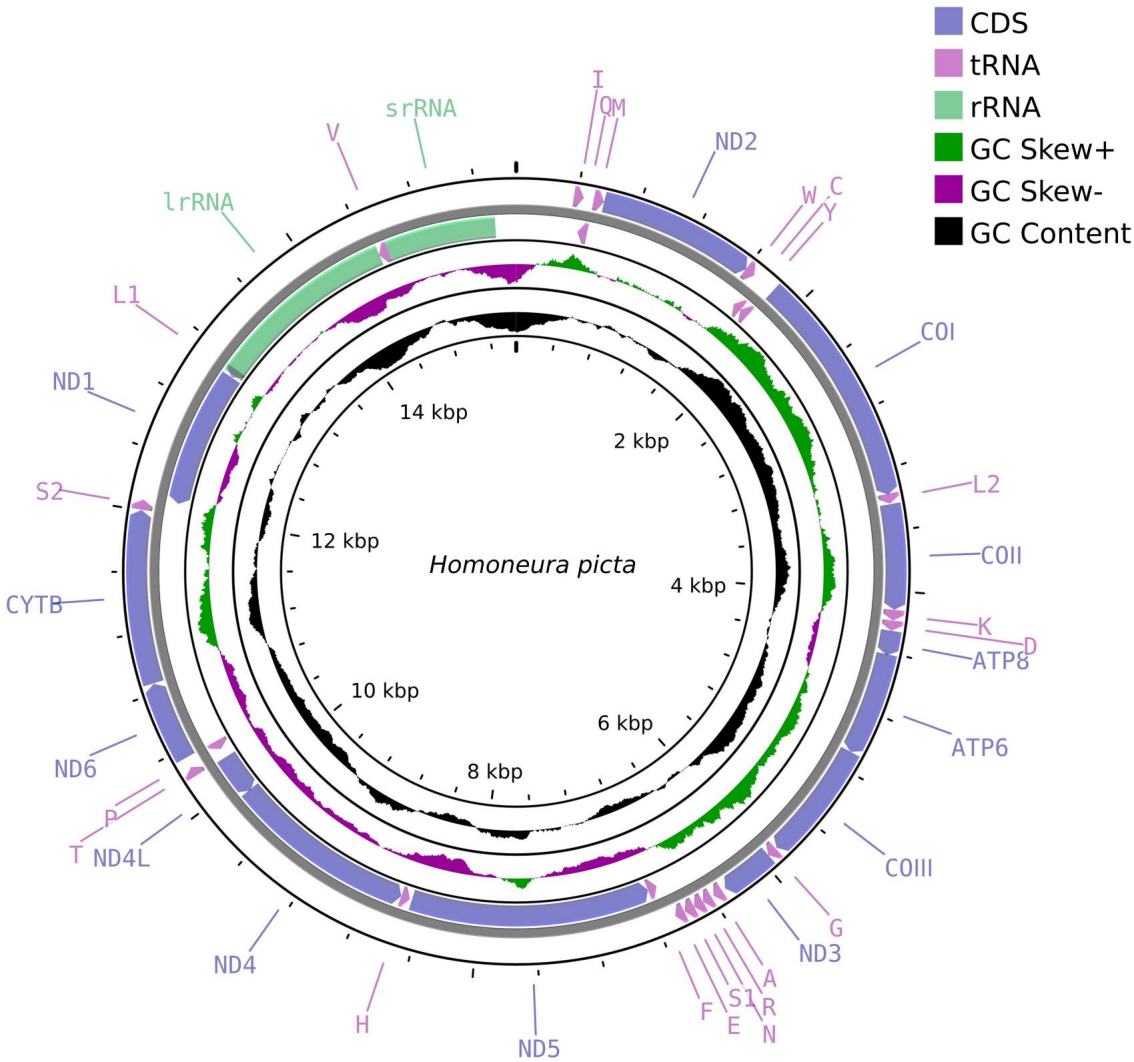
Mitochondrial genome map of *Homoneura picta*. Arrows indicate the orientation of gene transcription. Protein coding (CDS), tRNA, and rRNA genes are marked with different colors. The tRNAs are labeled according to the IUPAC-IUB single-letter amino acid codes. A GC-skew plot was created based on the deviation from the average GC-skew of the entire sequence. The GC content was plotted using a black sliding window, as the deviation from the average GC content of the entire sequence.

The approximate complete mitochondrial genome length of *H. picta* (Figure 2) was 15,469 bp, with a base composition of 38.4% A, 37.7% T, 14.1% C, and 9.8% G, showing distinct AT bias. It contained 37 genes typically found in insect mitochondrial genomes. Owing to the variable sequence and high A + T content of the control region, it was particularly difficult to characterize and we were unable to obtain the complete control region sequence. Despite belonging to a different genus, all genes of *H. picta* exhibit similar functions and locations to other known Homoneurinae genomes. Among the PCGs, twelve genes (*COII*, *ATP6*, *COIII*, *NAD4*, *NAD4L*, and *CYTB*) contained the start codon ATG, whereas the *COI* gene started with ACG. Most PCGs contained the stop codon TAA, while *COII* and *NAD4* contained T + trna as the stop codon. The 22 tRNA genes ranged from 62 to 72 bp in length, and all tRNA genes exhibited the typical clover-leaf secondary structure except for trnS1 (AGN), wherein the DHU arm was replaced by a loop. The 12S rRNA and the 16S rRNA genes were 787 bp and 1368 bp long, respectively (Table 1).

**Table 1.** Organization of the mitogenome of *Homoneura picta.*

Gene	Direction	Location	Size(bp)	**IGN** ^a^
tRNA^Ile^	F	377–441	65	
tRNA^Gln^	R	439–507	69	−3
tRNA^Met^	F	507–575	69	−1
*ND2*	F	576–1601	1026	0
tRNA^Trp^	F	1600–1667	68	−2
tRNA^Cys^	R	1682–1743	62	14
tRNA^Tyr^	R	1744–1809	66	0
*CO1*	F	1825–3366	1542	15
tRNA^Leu(UUR)^	F	3362–3426	65	−5
*CO2*	F	3431–4118	688	4
tRNA^Lys^	F	4119–4188	70	0
tRNA^a^^sp^	F	4189–4255	67	0
*ATP8*	F	4256–4417	162	0
*ATP6*	F	4411–5088	678	−7
*CO3*	F	5088–5876	789	−1
tRNA^Gly^	F	5885–5948	64	8
*ND3*	F	5949–6302	354	0
tRNA^a^^la^	F	6328–6391	64	25
tRNA^a^^rg^	F	6408–6470	63	16
tRNA^a^^sn^	F	6471–6536	66	0
tRNA^Ser(AGN)^	F	6537–6604	68	0
tRNA^Glu^	F	6608–6672	65	3
tRNA^Phe^	R	6706–6771	66	33
*ND5*	R	6772–8493	1722	0
tRNA^His^	R	8509–8573	65	15
*ND4*	R	8574–9912	1339	0
*ND4L*	R	9906–10,202	297	−7
tRNA^Thr^	F	10,205–10,269	65	2
tRNA^Pro^	R	10,270–10,335	66	0
*ND6*	F	10,338–10,862	525	2
*CYTB*	F	10,866–12,002	1137	3
tRNA^Ser(UCN)^	F	12,005–12,071	67	2
*ND1*	R	12,088–13,074	987	16
tRNA^Leu(CUN)^	R	13,076–13,139	64	1
lrRNA	R	13,096–14,463	1368	−44
tRNA^Val^	R	14,462–14,533	72	−2
srRNA	R	14,533–15,319	787	−1

^a^IGN: Intergenic nucleotide, minus indicates overlapping between genes.

**Figure 3.**
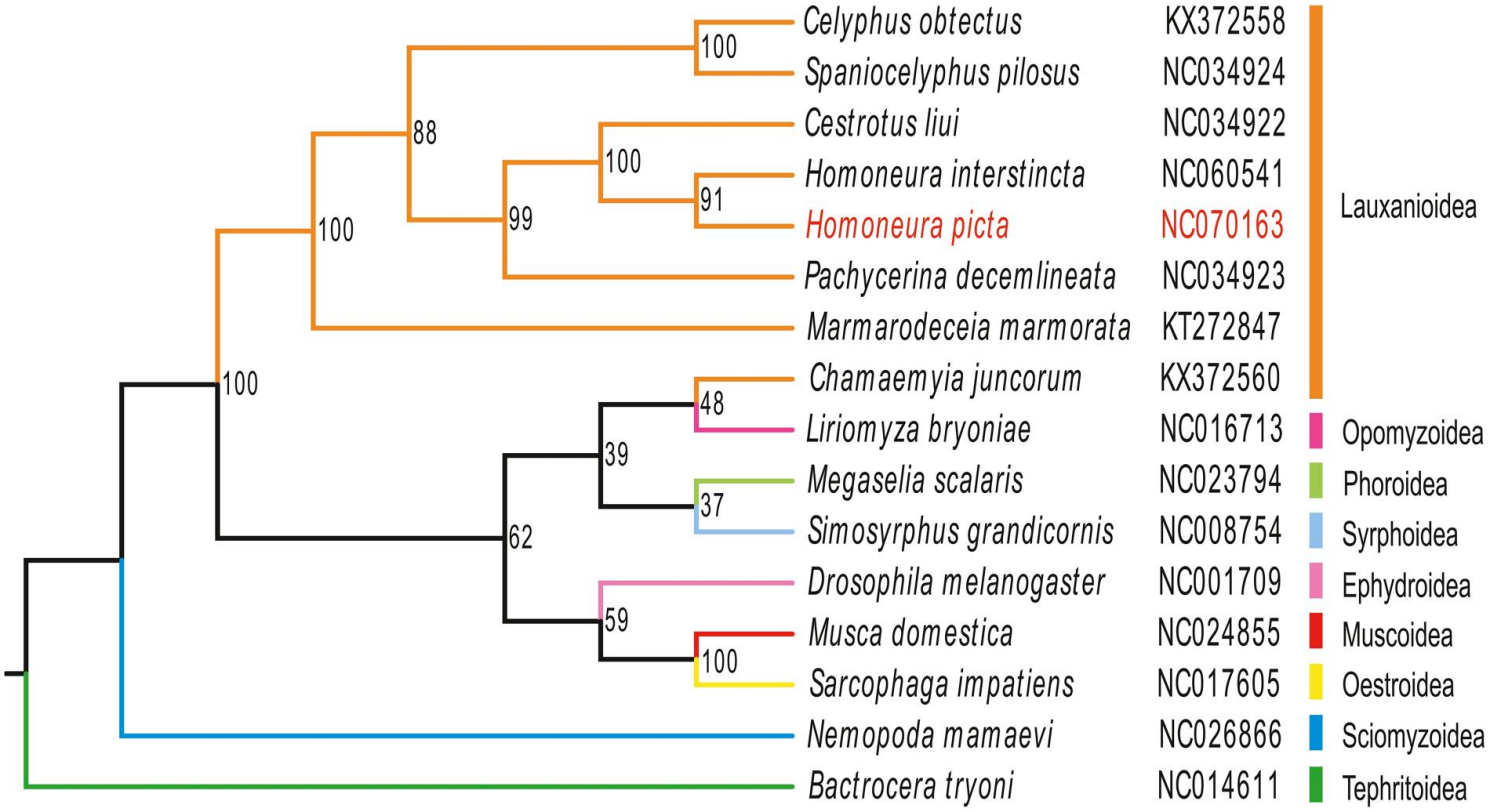
The phylogenetic tree was developed using the maximum-likelihood method, based on 13 PCGs and 2 ribosomal genes in the mitochondrial genome; the third locus of the mitochondrial genes was removed through nucleotide sequencing. The following sequences were used: NC060541 (unpublished), NC070163 (this study), NC034922, NC034923, KX372558, NC034924 (Li et al. 2017), KT272847 (Junqueira et al. 2016), NC014611 (Nardi et al. 2010), NC026866 (Li et al. 2015), NC001709 (Lewis et al. 1995), NC024855 (Li et al. 2016), NC017605 (Nelson et al. 2012), KX372560 (Li et al. 2017), NC023794 (Zhong et al. 2016), NC016713 (Yang et al. 2013), NC008754 (Cameron et al. 2007).

The Maximum likelihood tree indicated that *H. picta* belongs to the Lauxanioidea and is close to the *Homoneura* species *H. interstincta* (Figure 3). The phylogenetic relationship within Lauxanioidea was clear: Lauxanioidea was not monophyletic. Lauxaniidae was not a sister to the Chamaemyiidae family. However, Phoridae was a sister to Chamaemyiidae. *Homoneura* was a sister genus of *Cestrotus*.

## Discussion and conclusion

We described the first approximate complete mitochondrial genome of *H. picta*. The topology of the maximum likelihood tree was supported by morphological taxonomy. These results imply the application prospect of mitogenomic data in species taxonomy. *Homoneura* is the most diversified family of extant Lauxaniidae; however, its classification is still primarily based on morphological characteristics, and its ontogeny remains controversial owing to the lack of genomic information. Considering this, the mitochondrial genome of *H. picta*. reported in this study contributes to the understanding of the evolutionary relationships of this species. This work provides an essential DNA molecular database for further evolutionary and molecular research, and we aim to resolve the phylogenetic status of *Homoneura* species with mitochondrial DNA markers, which require abundant, high-quality mitochondrial genomes from this genus in the future.

## Supplementary Material

Supplemental Material

## Data Availability

The genome sequence data that support the findings of this study are openly available in GenBank of NCBI at https://www.ncbi.nlm.nih.gov, reference number NC070163. The associated BioProject, SRA, and Bio-Sample numbers are PRJNA983226, SRR24922755, and SAMN35722764, respectively.
