# Supplementary figures and images for: The mitochondrial genome of Homoneura picta (Diptera: Lauxaniidae) and its phylogenetic analysis
Source: Mitochondrial DNA B Resour. 2024 Jun 24;9(6):828–31. doi: 10.1080/23802359.2024.2333560 (PMC11198121; doi:10.1080/23802359.2024.2333560)

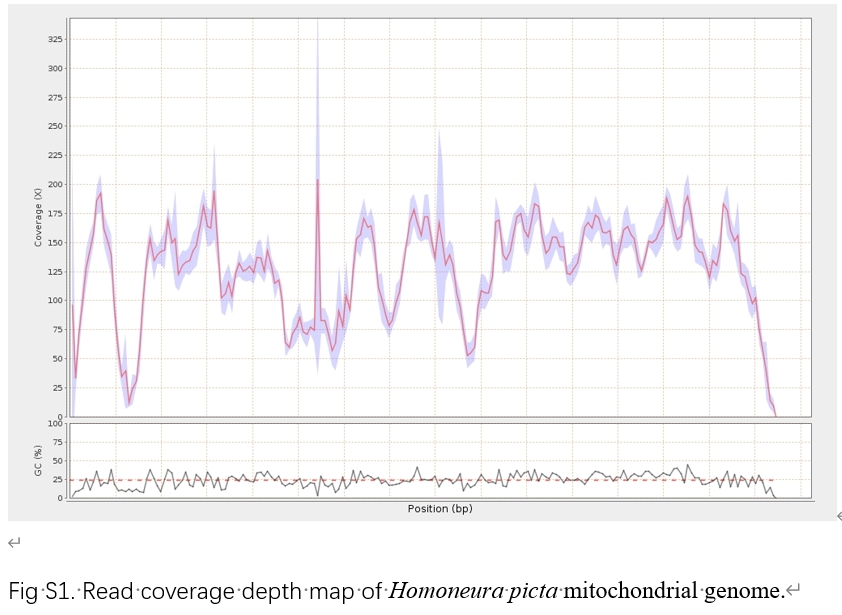

Supplement: Supplemental Material [file TMDN_A_2333560_SM1095.jpg]
